# Pseudomembranous Conjunctivitis: A Possible Conjunctival Foreign Body Aetiology

**DOI:** 10.7759/cureus.8176

**Published:** 2020-05-18

**Authors:** Dawn Ho, Shandy Lim, Yeo Kim Teck

**Affiliations:** 1 Ophthalmology, Ministry of Health Holdings, Singapore, SGP; 2 Ophthalmology, Apple Eye Centre, Singapore, SGP

**Keywords:** conjunctivitis, pseudomembrane, foreign body

## Abstract

Pseudomembranous conjunctivitis is commonly associated with infections. However, the normal flora of the human conjunctiva has been proven to harbour many of the microorganisms presumed to cause infective pseudomembranous conjunctivitis. We aimed to evaluate firstly the aetiology of pseudomembranes, and secondly the treatment of pseudomembranes. This case series exhibited foreign body as a common factor in the formation of pseudomembranous conjunctivitis requiring repeated foreign body and pseudomembrane removal with an early, sustained course of topical steroid for effective recovery.

## Introduction

Pseudomembranous conjunctivitis is an inflammatory condition of the conjunctiva characterised by conjunctival injection, mucopurulent discharge and pseudomembrane formation. A pseudomembrane is formed when inflammatory exudate rich in fibrin coagulates on the conjunctiva. This is seen as a thin yellow-white membrane in the fornices and palpebral conjunctiva that can be readily peeled off, leaving an intact underlying epithelium with minimal bleeding.

The formation of conjunctival pseudomembrane may be attributed to various causes. Infective causes, including *Corynebacterium diphtheriae*, *Neisseria gonorrhoeae*, *Streptococcus pyogenes* and adenovirus, are commonly reported [1]. Other aetiologies that have been reported include acute Stevens-Johnson syndrome and ligneous conjunctivitis, allergic or toxic factors such as gentamicin toxicity, and chemical irritants as well as vegetable and animal irritants [1-3].

One interesting and less commonly mentioned cause of pseudomembranous conjunctivitis is the presence of foreign body. Foreign body as a cause of conjunctival pseudomembrane formation was first reported in 1971 [2]. In this study, three cases of conjunctival pseudomembrane were sent for histological examination and found to demonstrate foreign body cellular reaction [1]. Of note, two of three of the conjunctival cultures also grew *Staphylococcus aureus*, while the third conjunctival culture was negative. The question arises as to the relevance of these foreign body findings in view of the positive *Staphylococcus aureus* cultures. Four points are proposed to answer this. Firstly, there are innumerable cases of conjunctivitis due to *Staphylococcus aureus* that do not form any membrane. Secondly, *Staphylococcus aureus* is commonly part of the normal conjunctival flora. A large population study of 10,271 patients in 1972 had noted a 42% frequency of *Staphylococcus aureus* colonisation [4]. Interestingly, in one of the three cases, no membrane was found until after a cotton swab was applied on the conjunctiva, and then the foreign bodies were identified microscopically as cotton fibres. Lastly, these cases clearly demonstrated foreign body giant cells surrounding birefringent foreign bodies on histology.

We present a case series of 12 eyes in nine patients with foreign body associated pseudomembranous conjunctivitis who were effectively treated with foreign body removal, membrane peeling and a course of topical steroid. To our knowledge, this is the largest case series of foreign body causing conjunctival pseudomembrane formation.

## Case presentation

Between 2016 and 2019, 12 eyes of nine patients were seen in a clinic with pseudomembranous conjunctivitis.

The duration of symptoms prior to attendance at the clinic was an average of six days, and as long as 14 days (Table 1). Six eyes in three patients had been seen by general practitioners prior to attendance with five eyes receiving a course of antimicrobial, but with little improvement.

**Table 1:** Prior to treatment at the clinic GP: general practitioner, SD: standard deviation

	Duration of symptoms (days)	GP seen previously	Treatment given by GP
1	1	No	Not applicable
2	5	Yes	Antibiotic, steroid
3	5	Yes	Antibiotic, steroid
4	14	Yes	Unsure
5	4	No	Not applicable
6	2	No	Not applicable
7	10	No	Not applicable
8	10	No	Not applicable
9	6	Yes	Antibiotic
10	1	No	Not applicable
11	7	Yes	Antibiotic, antiviral
12	7	Yes	Antibiotic, antiviral
Mean ± SD (range)	6 ± 3.76 (1-14)		

On ocular examination with the slit lamp, besides pseudomembrane, foreign body was found in all 12 eyes.

Random pseudomembrane samples from three eyes were sent for histological examination with one sample showing microscopic foreign material. No attempts were made to send all the foreign bodies for histology or to identify the type or composition of the foreign bodies as the primary purpose of the clinic visits was to treat the pseudomembranous conjunctivitis.

The course of treatment is outlined in Table 2. All eyes except one eye were started on dexamethasone eye drop. All eyes underwent foreign body and membrane removal. All eyes needed multiple foreign body removal (100% of eyes), while the majority of eyes needed multiple membrane peeling (75% of eyes). All eyes were effectively treated with an average duration of treatment of 14 days.

**Table 2:** Course of treatment SD: standard deviation

Eye	Duration of treatment (days)	Membrane peeling (times)	Foreign body removal (times)	Steroid given
1	15	2	2	Refused
2	16	4	3	Yes
3	16	4	4	Yes
4	3	1	2	Yes
5	12	1	2	Yes
6	17	2	5	Yes
7	27	2	2	Yes
8	27	3	2	Yes
9	19	2	2	Yes
10	3	1	2	Yes
11	10	5	2	Yes
12	10	5	2	Yes
Mean ± SD (range)	14.58 ± 7.38 (3-27)	2.67 ± 1.43 (1-6)	2.42 ± 0.86 (2-5)	

We present a particular case, Subject 9, in detail.

**Figure 1:**
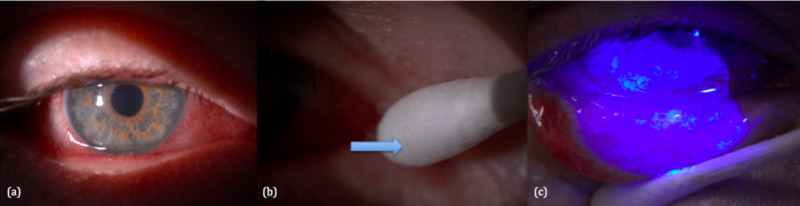
First review (12/01/2019): (a) injected conjunctiva, (b) foreign body removed, (c) pseudomembrane removed

A patient came to see us on 12/01/2019 because his right eye had been red and irritable for six days (Figure 1). He had seen a general practitioner on the first day and was given levofloxacin eye drop with little improvement. On examination, the bulbar and palpebral conjunctiva was injected with mucopurulent exudate on the inferior palpebral conjunctiva. Foreign body was found on slit lamp microscopy and removed from the inferior cul-de-sac. The pseudomembrane was peeled off, and he was started on dexamethasone eye drop.

**Figure 2:**
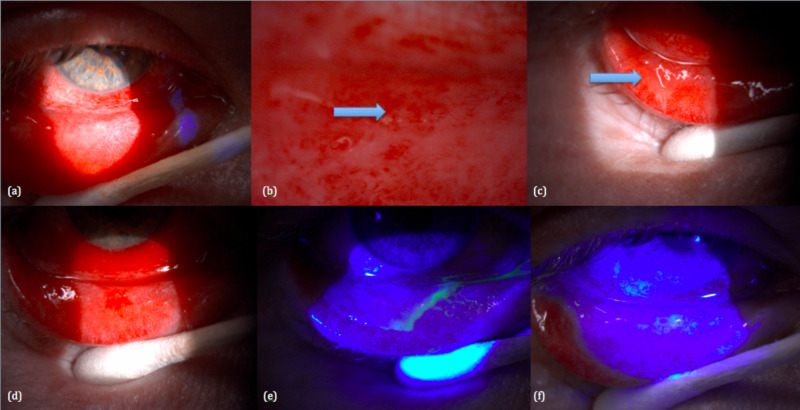
Second review (18/01/2019): (a) injected conjunctiva, (b) embedded foreign body, (c) pseudomembrane, (d) pseudomembrane removed, (e) fluorescein staining before and after pseudomembrane removal

When he came for his second review on 18/01/2019 (Figure 2), the conjunctiva was still injected with new pseudomembrane formation, highlighting the recurrent nature of pseudomembranes. This time, an embedded foreign body within the membrane was visible. Again, the foreign body was removed and the membrane was completely peeled off. We emphasise the importance of fluorescein staining in the ocular examination of patients with pseudomembranes. The pseudomembrane stains bright green prior to removal and the use of fluorescein clearly demonstrates its complete removal. He was kept on dexamethasone eye drop.

**Figure 3:**
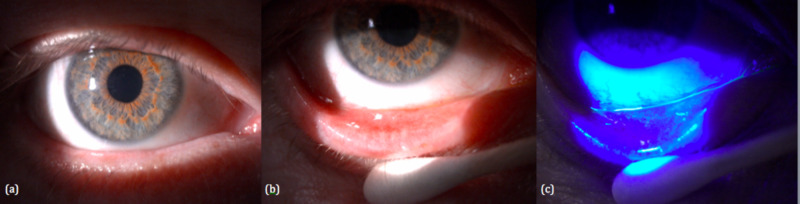
Third review (30/02/2019): (a-c) pseudomembranous conjunctivitis resolved

When he next saw us on 30/01/2019 (Figure 3), his pseudomembranous conjunctivitis had completely resolved. The conjunctiva was no longer injected, and there was no pseudomembrane found upon eversion of the eyelids and staining with fluorescein. The entire duration of his clinic visits was 19 days, although the conjunctivitis could have resolved earlier between his second and third visits. This case underscores the recurrent and possibly prolonged course of pseudomembranes, and hence the importance of a prompt and sustained course of steroid as well as repeated and complete membrane peeling and foreign body removal.

## Discussion

Pseudomembranous conjunctivitis has long been thought to be commonly linked to infections. The presumed infective aetiology of pseudomembranous conjunctivitis may be traced back to previous studies that have published the isolation of organisms, such as *Streptococcus*, *Staphylococcus*, *Klebsiella*, *Chlamydia*, *Corynebacterium *and adenovirus from the pseudomembranes of pseudomembranous conjunctivitis [5-8]. However, published studies of infective pseudomembranous conjunctivitis are few with most of these being case reports.

Moreover, it is of note that the bacteria that have been implicated in pseudomembranous conjunctivitis have also been established as part of the normal conjunctival flora in large case series and population studies. The presence of microorganisms in the normal human conjunctiva was established in the 19th century and has since been studied by numerous authors from various countries [4]. These studies have shown remarkable consistency across time and geography. From these studies, certain conclusions can be drawn.

Approximately 75%-82% of conjunctival cultures using conventional culture techniques have been found to be positive for at least one organism [9]. Coagulase-negative staphylococci is the most commonly found bacteria, detected in up to 100% of positive conjunctival cultures taken from patients, with *Staphylococcus epidermidis* being the predominant species [9]. This has been extensively corroborated in numerous studies since 1954 with studies coming from the USA, Japan, Korea, Finland, Uganda and even the rural populations of Sierra Leone [9]. Other organisms commonly constituting part of the ocular flora are *Staphylococcus aureus*, *Streptococcus *species, *Klebsiella *species, *Corynebacterium*, *Propionibacterium*, *Pseudomonas aeruginosa *and *Haemophilus influenzae* [4,9].

Conjunctival foreign body is probably more common than we expect, and foreign body induced pseudomembranous conjunctivitis may be more prevalent than we think. The conjunctiva may represent a locus minoris resistentiae with frequent exposure to external irritants such as dust or small foreign bodies [10]. These irritants may then initiate or perpetuate local inflammation and formation of pseudomembranes [2].

The mainstay of treatment of pseudomembranous conjunctivitis is pseudomembrane removal and treating the underlying cause, which includes antimicrobial cover of oft-presumed infective causes [1,2]. Our case series suggests the role of topical steroid and foreign body removal as adjuvants to pseudomembrane removal in the management of foreign body associated pseudomembranous conjunctivitis.

We noticed the intense inflammatory plus recurrent nature of pseudomembranes and targeted these two aspects in the treatment regime. We are of the opinion that a sustained course of steroid to dampen the inflammatory cascade, as well as repeated removal of the inflammation and the inflammatory trigger, namely the pseudomembrane and the foreign body, is effective as was our experience in our case series.

The need for repeated membrane peeling and foreign body removal highlights firstly the intense inflammatory nature of pseudomembranes, and secondly the common finding of foreign body as the inflammatory trigger either directly causing the pseudomembrane formation or acting as inciting agents superimposed upon underlying pathogens [2]. The necessity for a sustained course of steroid, repeated membrane peeling and foreign body removal in treating this condition is supportive evidence of the aetiology and inflammatory process.

There was one patient who was offered but refused steroid. We gathered on slit lamp photographs that he first presented with rather mild and limited pseudomembrane covering only one-third of his left lower lid. The pseudomembrane, although initially thin, mild and limited, was visibly still present after 15 days. That was the last time we saw him in the clinic, and he was subsequently lost to follow-up.

The span of symptoms and minimal improvement with only antimicrobial prior to attendance at the clinic further elucidates the role of membrane and foreign body removal with prompt commencement of steroid to alleviate symptoms and hasten recovery. Two eyes in our case series needed four pseudomembrane peeling procedures, each highlighting the intense nature of such inflammation requiring repeated membrane removal in addition to the use of steroid eye drops.

## Conclusions

In clinical practice, it is not unusual to encounter cases of pseudomembranous conjunctivitis that are particularly recurrent or long-standing and difficult to treat. Affected patients are frequently in severe and prolonged distress with very uncomfortable eye redness, swelling and discharge. The aetiology and treatment of pseudomembranous conjunctivitis may be more than meets the eye. The lookout for foreign body in the presence of pseudomembranes could pave the way for a more targeted, rapid and effective recovery, and better patient outcome and satisfaction.

## References

[1] Sahay P, Nair S, Maharana PK, Sharma N (2019). Pseudomembranous conjunctivitis: unveil the curtain. BMJ Case Rep.

[2] Norton AL, Green WR (1971). Foreign bodies as a cause of conjunctival pseudomembrane formation. Br J Ophthalmol.

[3] Bullard SR, O'Day DM (1997). Pseudomembranous conjunctivitis following topical gentamicin therapy. Arch Ophthalmol.

[4] Singer TR, Isenberg SJ, Apt L (1988). Conjunctival anaerobic and aerobic bacterial flora in paediatric versus adult subjects. Br J Ophthalmol.

[5] Janin A, Facon T, Castier P, Mancel E, Jouet JP, Gosselin B (1996). Pseudomembranous conjunctivitis following bone marrow transplantation: immunopathological and ultrastructural study of one case. Hum Pathol.

[6] Doddaiah V, Padmini H R, Seenivasen S, Vikram M (2014). Pseudomembranous conjunctivitis caused by Staphylococcus aureus. J Acad Clin Microbiol.

[7] Boparai MS, Dash RG, Ahmed KA (1983). Pseudomembranous conjunctivitis caused by Klebsiella. Afr Asian J Ophthalmol.

[8] Kluever HC (1935). Streptococcal pseudomembranous conjunctivitis: report of a case. Am J Ophthalmol.

[9] Grzybowski A, Brona P, Kim SJ (2017). Microbial flora and resistance in ophthalmology: a review. Graefes Arch Clin Exp Ophthalmol.

[10] Schuster V, Seregard S (2003). Ligneous conjunctivitis. Surv Ophthalmol.

